# Integument *CYP* genes of the largest genome-wide cytochrome P450 expansions in triatomines participate in detoxification in deltamethrin-resistant *Triatoma infestans*

**DOI:** 10.1038/s41598-018-28475-x

**Published:** 2018-07-05

**Authors:** Andrea B. Dulbecco, Débora E. Moriconi, Gustavo M. Calderón-Fernández, Soledad Lynn, Andrés McCarthy, Gonzalo Roca-Acevedo, Jhon A. Salamanca-Moreno, M. Patricia Juárez, Nicolás Pedrini

**Affiliations:** 1 Instituto de Investigaciones Bioquímicas de La Plata (INIBIOLP), CCT La Plata Consejo Nacional de Investigaciones Científicas y Técnicas (CONICET)-Universidad Nacional de La Plata (UNLP), La Plata, 1900 Argentina; 2 Centro Regional de Estudios Genómicos (CREG), Departamento de Ciencias Biológicas-Facultad de Ciencias Exactas-UNLP, La Plata, 1900 Argentina; 3 Centro de Investigaciones de Plagas e Insecticidas (UNIDEF-CONICET), Buenos Aires, B1603ALO Argentina

## Abstract

Insect resistance to chemical insecticides is attributed to a combination of different mechanisms, such as metabolic resistance, knockdown resistance, and the cuticular resistance or penetration factor. The insect integument offers an efficient barrier against contact insecticides and its role as penetration factor has been previously reported; however, there is no information about its potential function in the metabolic resistance. Cytochrome P450 genes (*CYP*) are highly expressed in the fat body of several insects and thus play a key role in their metabolic resistance. Here, we describe new members that belong to the highly genome-wide expanded *CYP3093A* and *CYP4EM* subfamilies in the Chagas disease vectors *Rhodnius prolixus* and *Triatoma infestans*. We modeled the docking of deltamethrin in their active site and detected differences in some amino acids between both species that are critical for a correct interaction with the substrate. We also knocked down the two constitutively most expressed genes in the integument of resistant *T*. *infestans* nymphs (*CYP3093A11* and *CYP4EM10*) in order to find clues on their participation in deltamethrin resistance. This is the first report on the role of the insect integument in detoxification events; although these two *CYP* genes do not fully explain the resistance observed in *T*. *infestans*.

## Introduction

Triatomine insects are vectors of the protozoan *Trypanosoma cruzi*¸ the causative agent of Chagas disease. *Triatoma infestans*, the main vector in South America, has been successfully controlled by pyrethroid residual spraying for over 30 years. However, several resistant foci were detected in the last decade, reaching more than 50 *T*. *infestans* populations mainly located in the Gran Chaco geographic region shared by Argentina, Bolivia and Paraguay^1,2^. An increased detoxification, reduced affinity of the site of action to the insecticide, and reduced penetration through the cuticle are the main resistance mechanisms described in insects to date^3,4^. These three mechanisms were already reported in deltamethrin-resistant *T*. *infestans*; i.e., increased activity of some detoxification enzymes, such as some cytochrome P450 monoxygenases and esterases^5,6^, point mutations in the sodium channel gene^7,8^, and a reduced insecticide penetration related to thickening of the cuticle together with larger amounts of cuticular hydrocarbons in the resistant insects compared to susceptible specimens^9,10^.

Cytochrome P450 monooxygenases constitute one of the largest superfamilies of enzymes found in nature, catalyzing the conversion of lipophilic compounds (either endogenous or xenobiotics) to more hydrophilic derivatives. Due to their large abundance, P450 genes (*CYP*) are assigned into families and subfamilies following a special nomenclature based on their amino acid sequence identity. A higher order for grouping *CYP* genes, called clan, has been applied to studies of P450s from the different domains of life^11^. P450s play a key role in detoxification of chemical insecticides; several *CYP* genes belonging either to *CYP3* or *CYP4* clans have been linked with insecticide resistance in Diptera^12^, Hemiptera^13^ and Coleoptera^14^. Expansions of *CYP* genes (“blooms”) in insect genomes are hypothesized to be a response to environmental stimuli that might drive the potential to acquire resistance to chemical insecticides^15^. Recently, a new *CYP* family belonging to the clan *CYP4* (*CYP3093*) was described in the Chagas disease vector *Rhodnius prolixus*^16^. The *CYP3093* family included the largest “bloom” detected in this species, particularly the subfamily *CYP3093A* showed 10 putatively functional genes (*CYP3093A1* to *A10*) plus 40 gene fragments dispersed throughout the entire genome. The second-largest P450 expansion described in this insect vector is formed by six *CYP4*-clan genes, *CYP4EM1* to *CYP4EM6*^17^.

The integument is the most external insect tissue; it is formed by the cuticle and the epidermis and plays an essential role in insect fitness and survival^18–20^. Although detoxification processes are recognized to occur in the insect fat body, contact insecticides find the integument as the first barrier to be surpassed prior to reaching the target site. A transcriptome analysis of *T*. *infestans* integument was recently performed reporting 57 *CYP* genes, most of them included in the *CYP3* and *CYP4* clans^21^. A suite of *CYP3*-clan genes was overexpressed (~1.7 to 3.8-fold) in resistant insects, being the first report of the potential detoxification role of the insect integument^21^.

In this study, we describe new *CYP4*-clan members that belong to the highly expanded *CYP3093A* and *CYP4EM* subfamilies in triatomines and also modeled the docking of deltamethrin to the active site of all clan CYP4 enzymes both in *R*. *prolixus* and *T*. *infestans*. We performed a differential expression analysis of these genes in the integument of pyrethroid-resistant *T*. *infestans* nymphs and investigated the induction of the *CYP3093A* and *CYP4EM* subfamilies by deltamethrin. Finally, we knocked down two constitutive genes that were expressed the most in the integument of resistant *T*. *infestans* nymphs (*CYP3093A11* and *CYP4EM10*) in order to find clues on their potential participation in deltamethrin resistance.

## Results

### Protein members of genome-wide *CYP* gene expansions fit deltamethrin better in *T*. *infestans* than in *R*. *prolixus*

**Figure 1:**
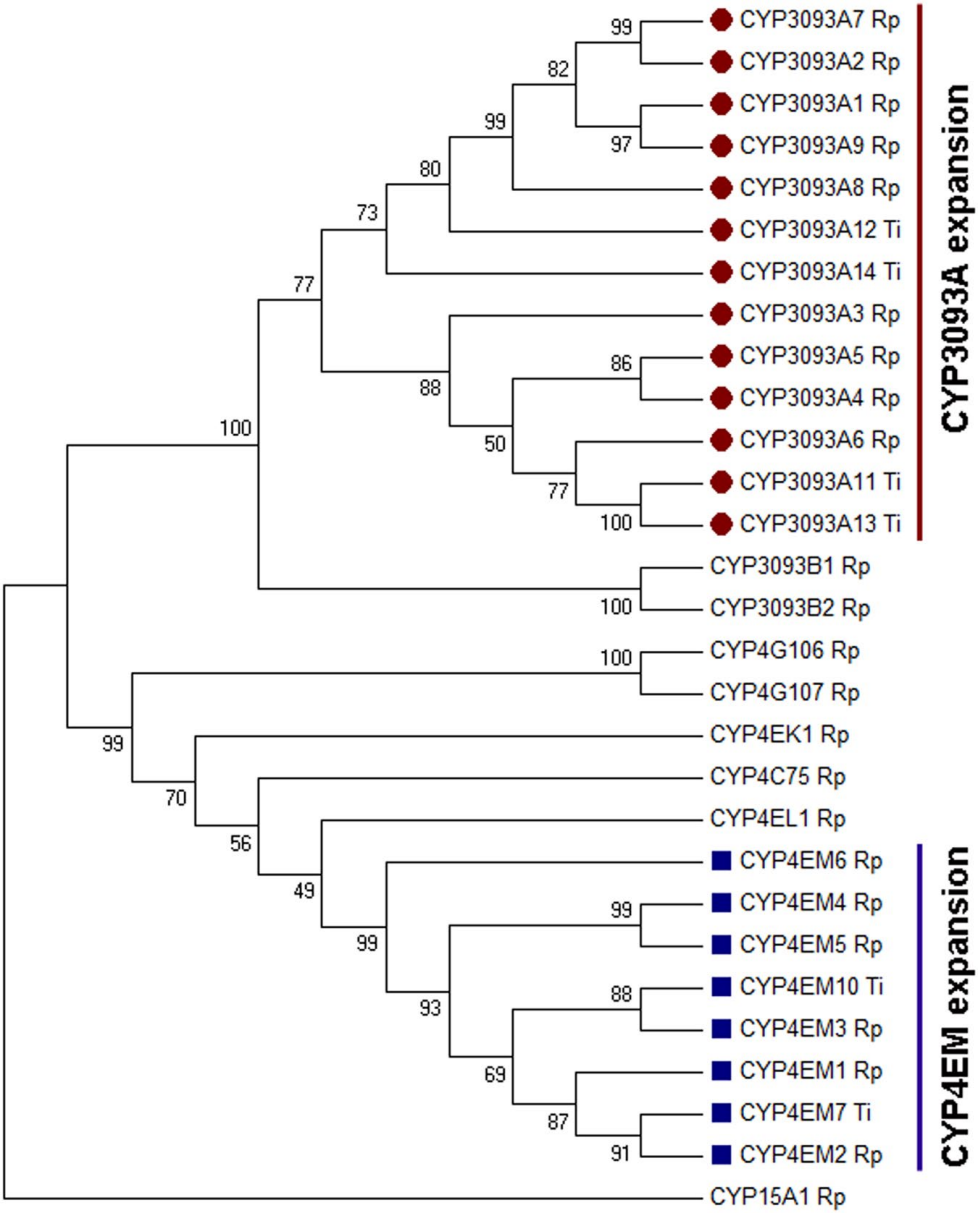
Maximum likelihood phylogenetic tree constructed with genes from clan CYP4 in *R*. *prolixus* (Rp) and *T*. *infestans* (Ti). Gene expansions are highlighted in red (CYP3093A subfamily) and blue (CYP4EM subfamily). CYP15A1Rp (clan CYP2) was used as outgroup. Numbers at nodes indicate groups support as the percentage from 1000 bootstrap replicates.

By mining into the *T*. *infestans* transcriptome libraries available^21,22^, we described new gene members belonging to the two largest CYP expansions in triatomines, *CYP3093A* and *CYP4EM*. After obtaining the full-length sequences, the new proteins were named by the P450 nomenclature committee as follows: CYP3093A11, CYP3093A12, CYP3093A13, CYP3093A14, CYP4EM7 and CYP4EM10. The evolutionary history of the clan CYP4 in triatomines was inferred using the maximum likelihood method. The phylogenetic tree (Fig. 1) shows that each expansion forms a distinctive clade, and CYP members from both insects were mixed up in each gene-expansion-representing clade.

**Figure 2:**
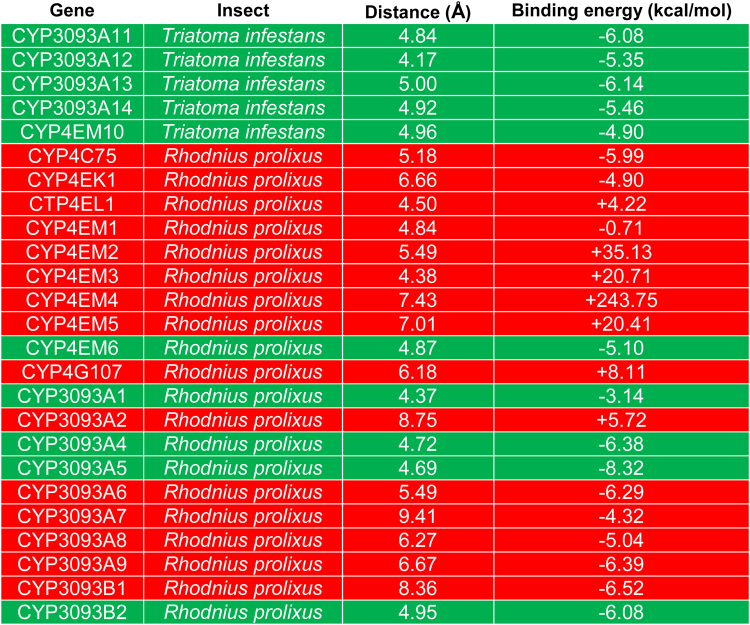
Molecular docking of deltamethrin at the active site of modeled enzymes belonging to clan CYP4 in *R*. *prolixus* and subfamilies CYP4EM and CYP3093A in *T*. *infestans*. The docking parameters were set as is described in Methods: distance ≤5 Å and binding energy ≤−2.39 kcal/mol. The green panels show cytochrome P450s that meet both interaction criteria and the red panels show enzymes that failed to meet at least one of these conditions.

We modeled the docking of deltamethrin to the enzyme-active site in all expanded members using the Autodock software. Because the most probable pathway for deltamethrin metabolism in insects is the 4′ carbon-hydroxylation^23,24^, we searched for an appropriate interaction looking into two different parameters: the distance between C4′ and the heme iron atom (should be 5 Å or less in order to guarantee hydroxylation), and the binding energy between deltamethrin and a cubical zone (20 × 20 × 20 Å) centered in the heme iron (should be within the 95% threshold of total negative binding energies). Figure 2 shows these results for all the enzymes; green panels represent P450s that were able to meet both interaction criteria with delthametrin, and red panels show enzymes that failed to meet at least one of these conditions. Thus, a distinctive behavior for *CYP4*-clan genes is evident for both species; while 100% of the *T*. *infestans* proteins exhibited a favorable docking between the substrate and the active site, only 25% of the *R*. *prolixus* proteins met these requirements (Fig. 2).

**Figure 3:**
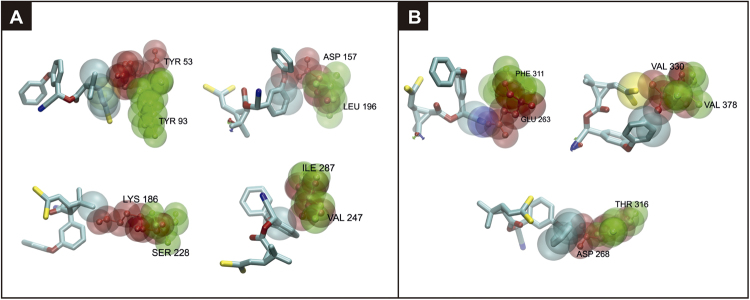
Changes observed in specific amino-acid residues from the active site pocket for each pair of cytochrome P450 in *T*. *infestans* (green) and *R*. *prolixus* (red). (**A**) Pair 1: CYP3093A11 (*T*. *infestans*) and CYP3093A6 (*R*. *prolixus*). (**B**) Pair 2: CYP4EM10 (*T*. *infestans*) and CYP4EM3 (*R*. *prolixus*).

Two phylogenetically close proteins, i.e., CYP4EM10 (*T*. *infestans*) and CYP4EM3 (*R*. *prolixus*), as same to CYP3093A11 (*T*. *infestans*) and CYP3093A6 (*R*. *prolixus*) (Fig. 1), showed different docking with deltamethrin (Fig. 2). In order to further elucidate the structural traits that could be responsible for these differences, we analyzed the amino-acid residues within the active site pocket for each cytochrome P450 pair, selecting those traits more apt to prevent an appropriate deltamethrin docking. CYP3093A11 and CYP4EM10 of *T*. *infestans* show either neutral or polar amino acids, but no charged residues, close to the deltamethrin molecule. Remarkably, their counterparts in *R*. *prolixus*, CYP3093A6 and CYP4EM3, contain ionic (either positively or negatively charged) residues (Table 1). We also observed that, although certain amino acids of the pocket are conserved within the protein sequence, their spatial disposition is not. These results show that the spatial disposition of the *T*. *infestans* P450 proteins modeled can accommodate substrate correctly, but the potentially steric effect predicted for *R*. *prolixus* P450s (Fig. 3) prevents suitable interaction with the substrate.

**Table 1:** Differences in specific amino acids of the active site pocket between two phylogenetically-related proteins from *Triatoma infestans* and *Rhodnius prolixus*.

**Pair 1**	**CYP3093A11 (*T***. ***infestans*)**	**CYP3093A6 (*R***. ***prolixus*)**
	ILE 287	VAL 247
	SER 228	LYS 186
	TYR 93	TYR 53
	LEU 196	ASP 157
**Pair 2**	**CYP4EM10 (*T***. ***infestans*)**	**CYP4EM3 (*R***. ***prolixus*)**
	THR 316	ASP 268
	VAL 378	VAL 330
	PHE 311	GLU 263

Two distinct pairs are compared.

### The integument of *T*. *infestans* exhibits P450-based detoxification ability

We measured the ethoxycoumarin-O-deethylase activity of P450 enzymes in the integument of resistant *T*. *infestans* nymphs by using a direct fluorometric method that quantifies the fluorescence of the reaction product, 7-hydroxycoumarin (7-OHC). We found that ethoxycoumarin was efficiently metabolized by this tissue, with an average activity value of 0.12 ± 0.02 pmol 7-OHC/min/integument.

### CYP3093A11 and CYP4EM10, but not their expansions, are the most expressed genes in the integument tissue of deltamethrin-resistant *T*. *infestans* nymphs

**Figure 4:**
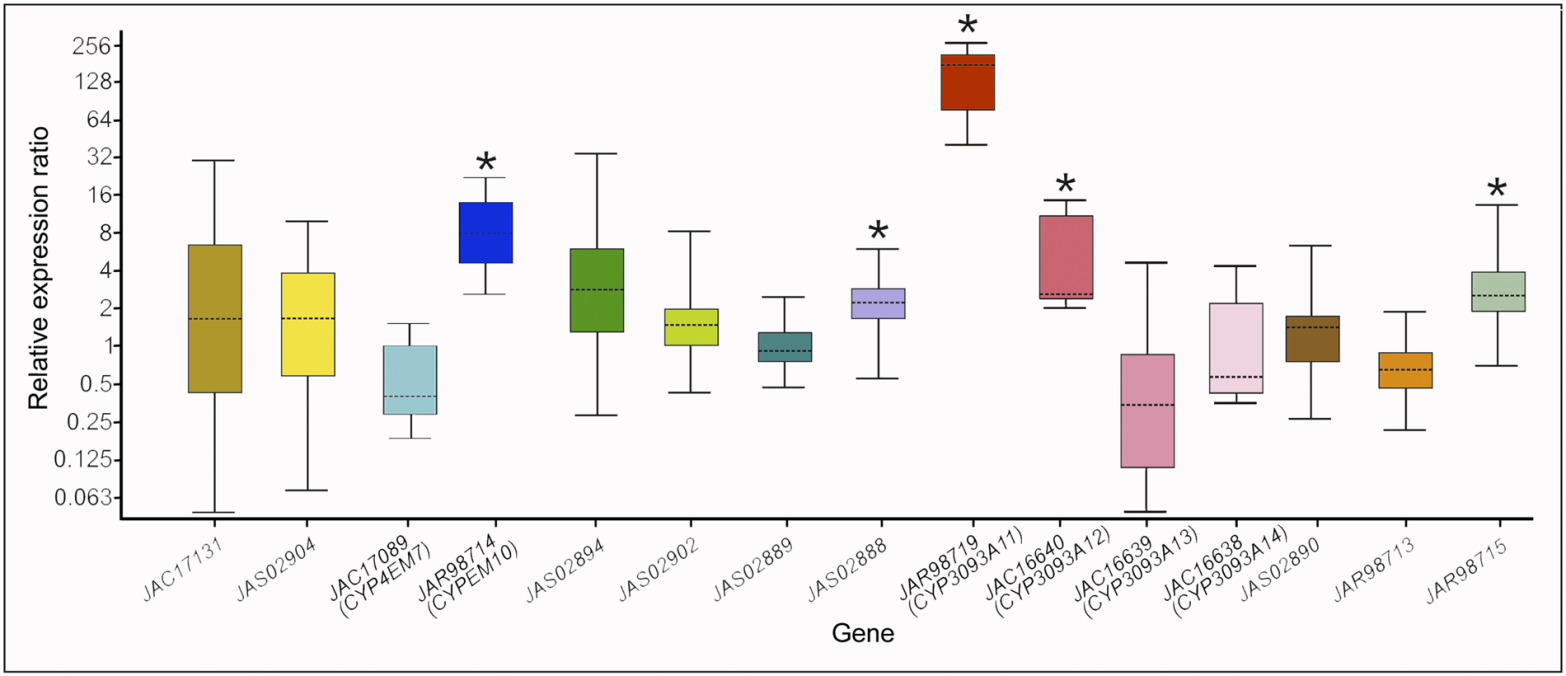
Relative expression analysis of *CYP4*-clan genes in the integument of 4th-instar *T*. *infestans* resistant nymphs compared to susceptible nymphs. The box area encompasses 50% of all observations, the dotted line represents the sample median of three biological replicates and the vertical bars represent the outer 50% of observations. Asterisks indicate significant differences (*p* < 0.05).

In a previous study, we identified 45 *CYP* gene fragments in a transcriptome analysis of *T*. *infestans* nymph integument^21^. Here, we performed a differential expression analysis by qPCR of the integument *CYP* genes classified under the clan *CYP4* (15 genes). Only five *CYP4*-clan genes were up-regulated (*p* < 0.05) in resistant nymphs (R) compared to susceptible nymphs (S) (Fig. 4), GenBank access and their transcription levels were: JAS02888 (2.2-fold expression), JAR98715 (2.6-fold expression), JAC16640 (4.3-fold expression), JAR98714 (7.6-fold expression) and JAR98719 (129.0-fold expression). Interestingly, the last three genes showed the highest differential expressions (corresponding to *CYP3093A12*, *CYP4EM10* and *CYP3093A11*, respectively) and are part of the gene expansions mentioned before. On the contrary, the other members of these expansions, *CYP3093A13*, *CYP3093A14*, and *CYP4EM7* were constitutively expressed at the same level in both R and S strains.

We also studied the expression pattern of these gene expansions in other tissues with well-known detoxification role in several insect orders; i.e. fat body, midgut, and Malpighian tubules. The most up-regulated genes in the integument of R nymphs showed no significant expression difference between R and S fat bodies, the values obtained were a 1.3-fold expression for *CYP3093A11* and 0.4-fold expression for *CYP4EM10*. Conversely, *CYP3093A12* and *CYP3093A13* were down-regulated (0.4- and 0.5-fold expression, respectively) and *CYP3093A14* were slightly more expressed (2.3-fold) in the fat body of R compared to S (Table 2). In the midgut and Malpighian tubules, half of the genes studied were not expressed both in S and R bugs; the rest of the genes were either down-regulated or not expressed in R nymphs compare with S insects (Table 2).

**Table 2:** Relative expression analysis for the expanded genes from *CYP3093A* and *CYP4EM* subfamilies in different tissues of resistant 4th-instar *T*. *infestans* nymphs, normalised with susceptible bugs (R/S).

Tissue	Gene					
	*CYP3093A11*	*CYP3093A12*	*CYP3093A13*	*CYP3093A14*	*CYP4EM7*	*CYP4EM10*
Integument	129,1 (59,6–204,1)*	4.3 (2.4–12.2)*	0.2 (0.1–1.0)	0.7 (0.3–2.4)	0.5 (0.3–1.2)	7.6 (4.0–14.4)*
Fat body	1.3 (0.5–3.7)	0.4 (0.2–0.7)*	0.5 (0.3–0.9)*	2.3 (1.1–4.5)*	0.9 (0.6–1.2)	0.4 (0.1–1.1)
Midgut	not expressed	0.5 (0.3–0.7)*	not expressed	not expressed	0.3 (0.2–0.3)*	0.5(0.3–0.9)*
Malpighian tubules	not expressed	1.3 (0.6–2.1)	not expressed	2.6 (0.9–7.6)	0.3 (0.1–0.6)*	not expressed

The values represent the sample median of four biological replicates and standard error in brackets represents the outer 50% of observations. Asterisks indicate significant differences (*p* < 0.05) between R and S insects.

A copy number variation (CNV) assay was performed by qPCR both in resistant and susceptible insects, in order to corroborate whether the high expression level observed in both *CYP3093A11* and *CYP4EM10* genes is due to constitutive overexpression in the integument, to a potential multiplication of specific DNA segments into the insect genome, or to both events. We found CNV’s ~1 from both target genes in both insect colonies; the data is reported in the Fig. S1 as the mean number of *CYP* copies ± standard error.

### Members of each *CYP3093A* and *CYP4EM* subfamilies show different time-course induction in the integument of resistant *T*. *infestans* nymphs after treatment with deltamethrin

**Figure 5:**
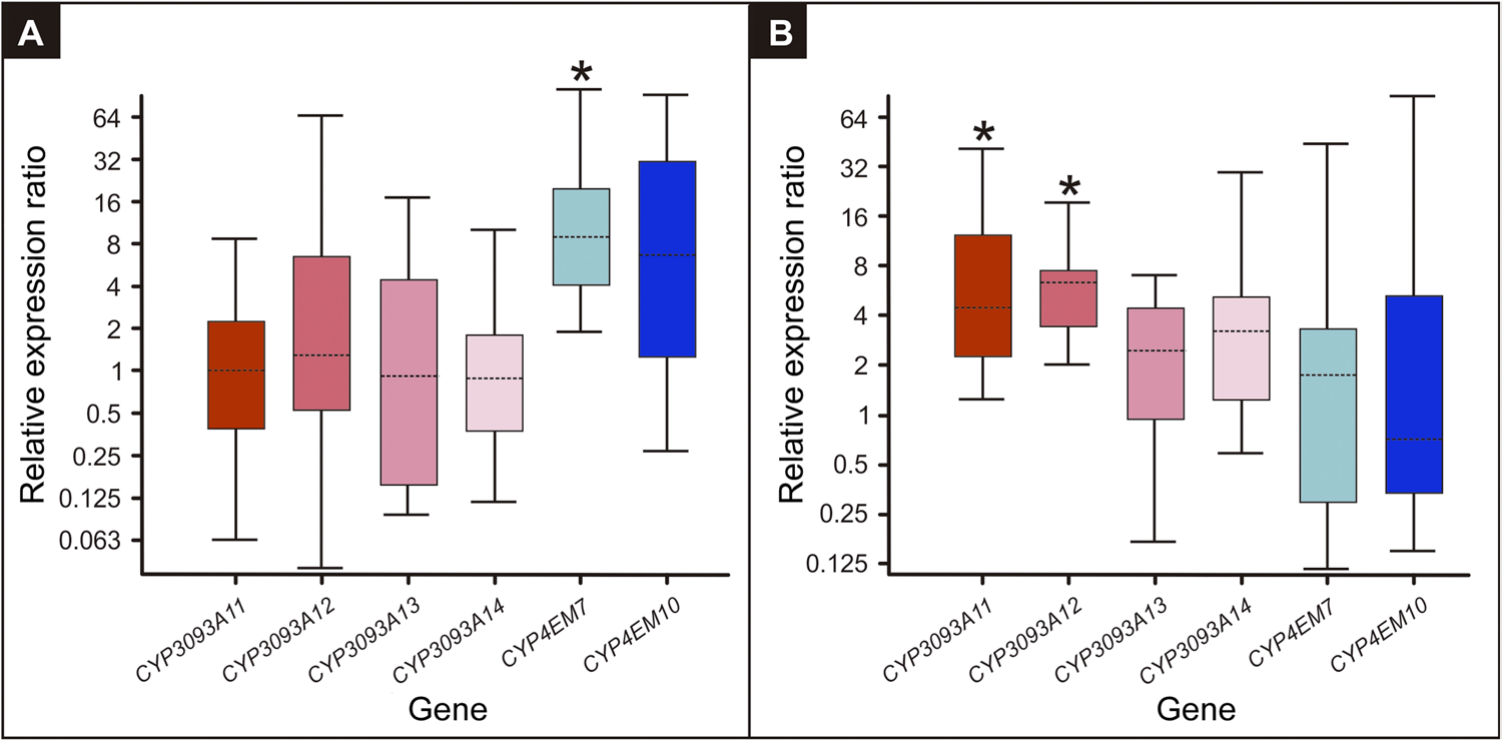
Induction of the expanded *CYP* genes by deltamethrin treatment. Boxplots show the gene expression of the subfamilies *CYP3093A* and *CYP4EM* at 3 h (**A**) and 72 h (**B**) after treatment of resistant 4th-instar *T*. *infestans* nymphs with deltamethrin at LD_50_ concentration. The box area encompasses 50% of all observations, the dotted line represents the sample median of three biological replicates and the vertical bars represent the outer 50% of observations. Asterisks indicate significant differences (*p* < 0.05).

Gene induction was also assessed after treatment of R nymphs with deltamethrin at LD_50_ concentration. The transcript levels of each gene expansion varied differently among different insecticide exposure time frames. *CYP4EM7* showed a significant induction (9.0-fold, *p* < 0.002) 3 h after deltamethrin topical application compared to controls treated with acetone. *CYP4EM10* also showed a higher induction (5.8-fold) than that observed in controls, although not statistically significant (*p* > 0.05) (Fig. 5A). Also, two members of the *CYP3093A* expansion were significantly up-regulated (*p* < 0.05) 72 h after deltamethrin topical application compared to controls; i.e., *CYP3093A11* (4.9-fold induction) and *CYP3093A12* (5.6-fold induction) (Fig. 5B).

### Injection of specific-dsRNA (dsCYP3093A11 and dsCYP4EM10) to resistant *T*. *infestans* nymphs leads to knock-down all *CYP3093A* and *CYP4EM* expansions without showing a specific deltamethrin-treatment phenotype

A significant suppression (40%, *p* < 0.01) of *CYP3093A11* mRNA level was observed after injection of dsCYP3093A11 in R nymphs; also, the expression of the other members of this expansion was knocked down (45% for *CYP3093A12* (*p* < 0.03), 75% for *CYP3093A13* (*p* < 0.02) and 76% for *CYP3093A14* (*p* < 0.03)), the same as the expression of other *CYP4*-clan genes (Table 3). JAR98715 was the only gene overexpressed (13.1-fold) in dsCYP3093A11-injected insects. Regarding the injection of dsCYP4EM10, the expression of both *CYP4EM10* and *CYP4EM7* was significantly reduced by 87% (*p* < 0.02) and 85% (*p* < 0.01), respectively; whereas five *CYP4*-clan genes were also knocked down in these insects (Table 3). Three *CYP4*-clan genes (JAS02890, JAS02894 and JAC17089) were silenced both in dsCYP3093A11- and dsCYP4EM10-injected insects. Injection with dsCYP4EM10 did not affect the expression pattern of all the genes belonging to *CYP3093A* subfamily (Table 3).

**Table 3:** Relative expression analysis for CYP4-clan genes in the integument of resistant 4th-instar *T*. *infestans* nymphs after injecting dsCYP3093A11 and dsCYP4EM10 (RNAi’s), normalized with resistant nymphs injected with control double-stranded RNA.

Gene	RNAi treatment	
	dsCYP3093A11	dsCYP4EM10
JAR98719 (*CYP3093A11*)	0.6 (0.5–0.7)*	1.5 (0.7–3.7)
JAC16640 (*CYP3093A12*)	0.5 (0.4–0.7)*	1.0 (0.9–1.2)
JAC16639 (*CYP3093A13*)	0.2 (0.1–0.4)*	0.9 (0.7–1.1)
JAC16638 (*CYP3093A14*)	0.2 (0.1–0.3)*	0.3 (0.1–1.1)
JAC17089 (*CYP4EM7*)	0.5 (0.2–0.9)*	0.2 (0.1–0.6)*
JAR98714 (*CYPEM10*)	1.0 (0.4–2.6)	0.1 (0.0–0.2)*
JAC17131	1.2 (0.8–1.8)	0.1 (0.0–0.1)*
JAS02904	0.3 (0.1–0.7)*	0.3 (0.1–0.6)
JAS02894	0.4 (0.2–0.7)*	0.1 (0.0–0.2)*
JAS02902	0.3 (0.0–1.1)	0.4 (0.1–1.8)
JAS02889	0.5 (0.2–0.9)*	0.9 (0.7–1.1)
JAS02888	0.6 (0.4–0.9)	0.6 (0.4–1.0)
JAS02890	0.4 (0.3–0.5)*	0.7 (0.6–0.8)*
JAR98713	1.6 (0,6–4,4)	0.3 (0,1–0,5)*
JAR98715	13.1 (8.5–20.7)*	0.3 (0.3–0.4)*

The genes are named by their GenBank access number and also with the official CYP name when available. The values represent the sample median of three biological replicates and standard error in brackets represents the outer 50% of observations. Asterisks indicate significant differences (*p* < 0.05).

**Figure 6:**
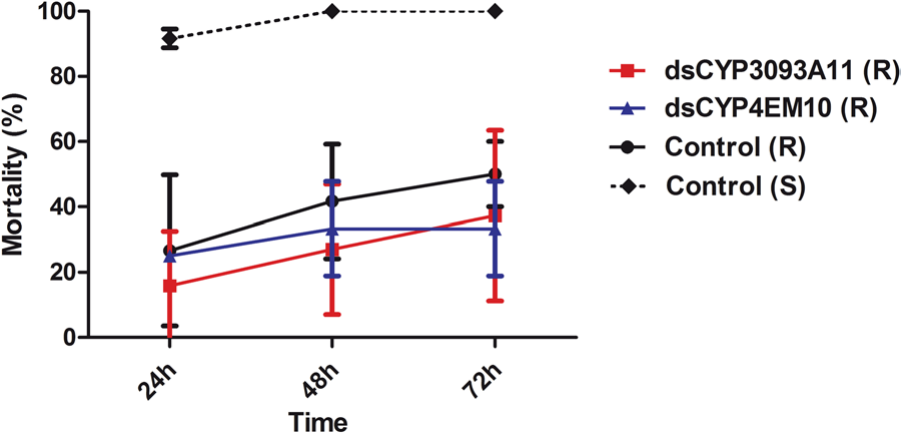
Mortality of dsRNA-treated nymphs after deltamethrin topical application at LD_50_ concentration of resistant insects. Prior to deltamethrin application, resistant insects were injected with dsCYP3093A11 (red), dsCYP4EM10 (blue), control double-stranded RNA (black solid line), and susceptible insects were injected with control double-stranded RNA (black dotted line).

In the presence of deltamethrin, the RNAi-induced phenotypes were not significantly different for R insects injected either with dsCYP3093A11 or dsCYP4EM10, compared to R control insects injected with non-related dsRNA. Figure 6 shows that the mortality of R control insects treated with deltamethrin at LD_50_ concentration continuously increased from 25% to 50% (at 24 h and 72 h, respectively) whereas the mortality observed for resistant nymphs injected with dsCYP3093A11 varied from 15% (24 h) to 35% (72 h). For dsCYP4EM10-injected insects, the mortality at 24 h was similar to that observed for R controls, reaching a plateau of 35% at 48 h. There were no significant differences between treatments (*p* > 0.05, two-way ANOVA). As expected, S insect mortality was above 92% mortality after 24 h.

## Discussion

Insecticide resistance in Chagas disease vectors is known to occur by a multiplicity of physiological-biochemical mechanisms acting in an overlapping manner. Regarding the metabolic resistance, the published biochemical studies were not able to explain the high resistance ratio values (100 < RR < 1000) observed in *T*. *infestans* populations from the Gran Chaco region, with no relevant differences detected in cytochrome P450, esterase (EST) and glutathione-S-transferase (GST) activities between R and S populations^6,25^. We have recently shown that expression induction of some *CYP3*-clan genes encoding for such detoxification enzymes was evident in the integument of R nymphs (RR = 100) although appeared to be not enough to support a significant participation in the resistance mechanism^21^. This study showed that cytochrome P450-based detoxification is active in *T*. *infestans* integument, as evidenced by its ethoxycoumarin-O-deethylase activity. The total activity here measured (0.12 ± 0.02 pmol 7-OHC/min/integument) was of the same order of magnitude than those reported in whole abdomens of 1st-instar nymphs (from 0.1 to 1.6 pmol 7-OHC/min/abdomen, depending on the population origin and large resistance ratio variability)^6^. Taking into account that the abdomens contain various tissues with detoxification activity, including the integument itself, we conclude that the values here reported allow endorsing P450 activity to the integument. Thus, this is the first report in insects on the role of the integument in detoxification processes; the molecular and bioinformatics approaches applied in the current study might help to further understand the mechanisms involved in metabolic resistance.

*CYP* expansions in insects are restricted to some subfamilies often associated with environmental responses (*CYP3* and *CYP4* clans) but not with those subfamilies with an endogenous function. This observation has led to propose the hypothesis of rapid birth-death evolution, differentiating *CYP* genes into phylogenetically stable and unstable. The stable genes have core functions in development and physiology, whereas the unstable genes have accessory functions associated with environmental interactions^26^. In this context, the insect *CYP3* and *CYP4* clans might have important roles on xenobiotic detoxification and hence drive the insect potential to acquire resistance to many chemical insecticides. Our study in *T*. *infestans* contributes to the knowledge of the major gene expansion detected in the *CYP4* clan described in the *R*. *prolixus* genome^17^ and is in agreement with the hypothesis that “blooms” in some specific *CYP*-subfamilies might be a common strategy in triatomines that triggers the emergence of insecticide resistance. The upcoming whole genomes of other species such as *T*. *infestans*, *T*. *brasiliensis* and other triatomines might help confirm this hypothesis. Taking into account the sequences known so far, our homology modeled results show that all *T*. *infestans CYP4*-clan proteins fit deltamethrin better than most proteins of the same CYP clan in *R*. *prolixus*. In this regard, we focused on the two most homologous pairs of proteins: CYP3093A11 (*T*. *infestans*) and CYP3093A6 (*R*. *prolixus*) as well as CYP4EM10 (*T*. *infestans*) and CYP4EM3 (*R*. *prolixus*).

*T*. *infestans* CYP3093A11 shows a binding site cavity with partial hydrophobic nature, due to the presence of two apolar-hydrophobic residues, i.e. ILE 287 and LEU 196. In the *R*. *prolixus* homologous counterpart (CYP3093A6), LEU 196 is replaced by ASP 157 (a negatively charged residue), thus reducing the capacity of the binding site to interact hydrophobically with deltamethrin. The other three relevant binding site residue differences (i.e. ILE 287 for VAL 247, SER 228 for LYS 186, and TYR 93 for TYR 53) correspond to spatial displacements interfering with the position in which deltamethrin is able to bind. Likewise, a similar trend of substitutions are observed after comparison of CYP4EM10 (*T*. *infestans*) versus CYP4EM3 (*R*. *prolixus*). Namely, a hydrophobic residue (PHE 311) is replaced by a negatively charged one (GLU 263), as well as a polar residue (THR 316) is replaced by a negatively charged one (ASP 268). Both substitutions result in a loss of capacity of the active site to bind deltamethrin hydrophobically. A third relevant binding site substitution (VAL378 for VAL330), i.e. the position of VAL330 within the binding site of CYP4EM3 creates a steric effect that prevents deltamethrin from binding correctly in order to achieve hydroxylation.

Although these proteins are phylogenetically close within each pair, specific differences in the amino acids of the active site between both species might contribute to a more efficient interaction and, finally, a favorable deltamethrin hydroxylation in *T*. *infestans*. This observation might be relevant because there is plenty information about deltamethrin resistance in different populations of *T*. *infestans*^2,5,6,27–32^; on the contrary, there is only one report of small resistance ratio in *R*. *prolixus*^33^. Focusing on the metabolic resistance phenomenon, we might speculate that both species could have expanded their *CYP4*-clan repertoire as an evolutionary strategy, although minor modifications in the amino acids of the active site—including the presence of ionic residues in *R*. *prolixus* compared to uncharged residues in *T*. *infestans*—might be responsible for large differences in deltamethrin interaction and thus metabolization by P450s.

*CYP* genes are known to be constitutively overexpressed in resistant insects from several orders^34^. In this study, we showed that only one gene of each *CYP* expansion has high constitutive expression levels in the integument of *T*. *infestans* resistant insects compared to susceptible specimens, namely *CYP3093A11* (130-fold) and *CYP4EM10* (8-fold). These genes, however, were not overexpressed in other tissues, such as fat body, midgut and Malpighian tubules. The other members of both expansions showed slight or no expression differences between S and R insects in all the tissues examined. In contrast with the constitutively high overexpression here reported for some members of the clan CYP4 in the integument of R specimens of *T*. *infestans*, the more numerous CYP3-clan genes showed a different behavior pattern; i.e., 4 out of 18 genes were significantly overexpressed in R compared to S nymphs, although showing moderate overexpression values ranging from 2- to 4-fold^21^. Grosso *et al*.^13^ employed a different qPCR approach (Taqman probe) to measure the expression of another *CYP4EM7* version with seven amino acids different from the gene here reported (probably representing a different allele or a splice variant) and two CYP3-clan genes (*CYP3085B1* and *CYP3092A6*) in the fat body of R insects. High expression levels were only reported for *CYP4EM7* in R nymphs, similar to those here reported in the integument for *CYP3093A11*. Regarding gene induction by deltamethrin at LD_50_ concentration, the relative expression values obtained 3 h after insecticide application for *CYP4EM7* in the integument were lower than those reported for the fat body *CYP4EM7*^13^. The other integument CYP genes here studied showed to be less inducible by deltamethrin than *CYP4EM7*. At longer times after insecticide exposition (72 h), *CYP3093A11* and *CYP3093A12*, but not *CYP4EM7* and *CYP4EM10*, were significantly induced by deltamethrin in the integument.

In an attempt to characterize the role of both *CYP3093A11* and *CYP4EM10* in deltamethrin detoxification, we injected specific-dsRNA into 4th-instar R nymphs. A reduced expression not only on the corresponding genes but also on other expansion members and other *CYP4*-clan genes was found. It is not surprising that expression of the entire expansion is suppressed because all expansion members share highly homologous sequences; similar results were reported for nitrophorins in *R*. *prolixus*^35^. However, we did not observe a clear phenotype after deltamethrin application in RNAi-injected insects. Because P450s are members of a large superfamily of enzymes, with almost 90 CYP genes described in *R*. *prolixus*, several members of each family or clan might perform similar or overlapping functions, e.g., in insecticide detoxification. This redundancy might lead in compensation to the loss of function of the targeted genes, masking the silenced phenotype as observed for both dsCYP3093A11 and dsCYP4EM10. In this sense, we found that one gene (JAR98715) out of all *CYP4*-clan genes studied was 13-fold induced in integuments of resistant bugs injected with dsCYP3093A11. This high expression level might act as a compensation response and thus be the responsible, at least in part, of the absence of a specific deltamethrin-treatment phenotype. This observation opens an exciting scenario for future studies; e.g., a potential crosstalk between both genes in the integument might have an active role, therefore, in deltamethrin metabolic resistance.

Furthermore, other CYP4-clan genes classified under the subfamily CYP4G were shown to be involved in the biosynthesis of cuticular hydrocarbons in *Drosophila melanogaster*^36^. The orthologous genes are overexpressed in resistant *Anopheles gambiae* also showing a significant increase in the hydrocarbon content of resistant mosquitoes^37^. This information also contributes to the hypothesis that not only multiple mechanisms are co-evolving in resistant insects but also that *CYP* genes exhibit manifold roles participating both in cuticular and detoxification resistance processes. Ongoing studies on the CYP4Gs in *R*. *prolixus* and *T*. *infestans* are expected to provide valuable information on this topic in triatomines.

## Conclusion

The main conclusion of the current study is that the P450-dependent detoxification is active in the integument of the Chagas disease vector *T*. *infestans*, suggesting a potential role in metabolic resistance. Genome-wide expansion of members of some CYP subfamilies that fit deltamethrin in their active site, both with negative binding energies and the appropriate distance and angle, might make efficient the degradation of deltamethrin. Also, the high rates in constitutive expression of specific members of these expansions in the integument of resistant insects, i.e., *CYP3093A11* and to a lesser extent *CYP4EM10*, ensure a high number of transcripts, which may be further increased in the presence of the substrate since these genes were shown to be inducible by deltamethrin. Thus, the involvement of the insect integument in the metabolic resistance phenomenon is proposed here for the first time, participating as an active barrier in insecticide detoxification prior to its transport to the traditionally recognized detoxification sites. However, a clear phenotype was not evident after attempting to silence their expression, indicating that these genes do not fully explain the resistance phenomenon. Other detoxification sites (fat body), detoxifying genes (*GST*, *EST*, and other *CYPs*) as well as other mechanisms, such as the cuticular penetration factor or altered action site, are involved, reinforcing the hypothesis of the multifactorial nature of insecticide resistance.

## Methods

### Gene mining and naming

All *R*. *prolixus CYP* sequences belonging to clan CYP4, including the genome-wide expansion of both *CYP4EM* and *CYP3093A* subfamilies, were obtained from the complete genome at VectorBase webpage (https://www.vectorbase.org/) using the gene ID provided by Schama *et al*.^17^. The sequences of CYP gene fragments of *T*. *infestans* previously classified under the CYP4 clan were obtained from expressed sequence tag (EST) libraries from the integument (GenBank, BioProject PRJNA314811)^21^ and salivary glands (GenBank, BioProject PRJNA238208)^22^. Sequences belonging to the subfamilies CYP4EM and CYP3093A were officially named by the P450 Nomenclature Committee (Dr. D. Nelson, personal communication) as follows: CYP4EM7 (GenBank JAC17089), CYP4EM10 (GenBank JAR98714), CYP3093A11 (GenBank JAR98719), CYP3093A12 (GenBank JAC16640), CYP3093A13 (GenBank JAC16639), and CYP3093A14 (GenBank JAC16638). Rapid amplification of cDNA ends (RACE) technique was used to obtain the full length of these gene fragments by using the FirstChoice® RLM-RACE kit (Ambion, Austin, USA), following the manufacturer’s instructions. Since this technique failed to obtain the 5′ end of both *CYP4EM10* and *CYP3093A11* sequences from *T*. *infestans*, we searched for the best alignment by using the alignment tool for protein sequences (SIM http://web.expasy.org/sim/) (Huang & Miller, 1991) to identify homologous sequences and then complete them with the best matching sequence. Thus, CYP4EM10 was completed with CYP4EM7 sequence while CYP3093A11 was completed with CYP3093A13 sequence.

### Phylogenetic analysis

The analysis involved 29 amino acid sequences, including the CYP15A1 (clan CYP2) from *R*. *prolixus* used as outgroup. All positions containing gaps and missing data were eliminated and then the sequences were aligned using Muscle^38^. The phylogenetic tree was constructed using the Maximum Likelihood method^39^ with 1000 bootstrap replications in MEGA 6.0 software^40^.

### Homology modeling

All sequences belonging to the clan CYP4 in both *R*. *prolixus* and *T*. *infestans* were homology modeled by using Phyre2 v2.0 online server, available at (http://www.sbg.bio.ic.ac.uk/)^41^. The server uses PSI-BLAST to find homologue templates in order to model the 3D structure of the provided sequence. The coordinates for the heme group were obtained from the template 1TQN and positioned as in the template of the homology modeled proteins using Swiss-PdbViewer (http://www.expasy.org/spdbv/). Molefacture Plugin v1.3 (from Visual Molecular Dynamics package) was used to build the thiol bond between the heme iron and the corresponding CYS sulfur atom for each protein. The model for CYP3093A3 (*R*. *prolixus*) was incompatible with the heme-cys thiol bond and thus did not fit for docking. All models were accessed with PROCHECK (http://services.mbi.ucla.edu/PROCHECK/) for geometric evaluation. Deltamethrin molecule (ZINC01532094) was retrieved from the library in ZINC12 database in MOL2 format (https://zinc.docking.org/).

### Molecular docking

Autodock Tools Version 4.2 was used to dock the ligand to the protein active site. Affinity maps of grids were calculated using AutoGrid program. Docking was carried out with the grid size set to 20 × 20 × 20 Å with 1 Å grid spacing and centered in the heme iron of each protein. The AutoDocking parameters used were of GA population size 150 and maximum number of energy evolutions 25,000,000. During docking, a maximum number of 10 conformers was considered, and the root-mean-square (rms) cluster tolerance was set to 2 Å. Models obtained for CYP4EM7 (*T*. *infestans*) and CYP4G106 (*R*. *prolixus*) were unable to dock.

Two concomitant conditions were established in order to analyze the deltamethrin-CYP interaction: *i*) the distance between C4′ and heme iron atom must be 5 Å or less, i.e., compatible with the hydroxylation of the phenoxybenzyl 4′ carbon that is the most probable route of deltamethrin metabolism in insects^23,24^, and *ii*) binding energy must come within the 95% threshold of total negative binding energies. Conformations with the appropriate interaction distance to the heme iron were selected and their interaction energy evaluated. The simulation results were illustrated using the Visual Molecular Dynamics package^42^.

### Insect rearing and tissue dissection

Fourth-instar nymphs of both deltamethrin susceptible (S) and resistant (R) *T*. *infestans* colonies were used. Insects were reared at the insectarium of the Instituto de Investigaciones Bioquímicas de La Plata (INIBIOLP) at 30 °C and 50–60% relative humidity under a photoperiod of 12:12 (L:D) h, and fed weekly on rats. Colonies are periodically renewed by incorporating first-generation insects, usually from Formosa province, provided by the Servicio Nacional de Chagas, Cordoba (S) and from Salta province, provided by Dr. R. Cardozo, National University Salta (R), both in Argentina. All animal care and laboratory experimental protocols were approved by the Directive Board of the INIBIOLP (Animal Welfare Assurance No. A5647–01) and carried out following the AVMA Animal Welfare Policies and AVMA Guidelines on Euthanasia: https://www.avma.org/kb/policies/pages/default.aspx, https://www.avma.org/KB/Policies/Documents/euthanasia.pdf, accessed 22 November 2017.

The abdomens of S or R nymphs were excised from the thorax and submerged in ice-cold saline solution (0.9% NaCl). Immediately, the fat body, midgut and Malpighian tubules were removed under a stereoscopic microscope (Zeiss Stemi 305, Oberkochen, Germany), and separately submerged and stored in RNAlater (Ambion, Austin, USA) until further use. The epidermis of the abdominal terguites and sternites was gently scrapped and the integuments were submerged either in 0.05 M phosphate buffer (for enzyme activity) or in RNAlater solution (for RNA extraction).

### Cytochrome P450 activity

P450 activity was measured in integuments dissected from R nymphs, by using 7-ethoxycoumarin (7-EC) (Sigma-Aldrich, St. Louis, MO, USA) as substrate, according to the direct fluorometric test method reported for individual abdomens of *T*. *infestans*^6^ with minor modifications. This method is based in the ethoxycoumarin-O-deethylase activity of P450 enzymes through quantification of the fluorescence of the reaction product, 7-hydroxycoumarin (7-OHC). Six integuments were pooled in phosphate buffer and placed into each well from a 96-well micro-plate containing 0.05 M phosphate buffer and 4 mM 7-EC (total volume 100 µl). The reaction was stopped after 4-h incubation at 30 °C by adding 100 µl of glycine buffer (10^−4^M), pH 10.4. To precipitate the integuments in the wells, the plates were centrifuged at 2,000 × g for 30 s in a refrigerated centrifuge (4237 R, ALC International SRL, Cologna Monzese, Italy). For each condition, similar wells receiving glycine buffer before incubation were used as blanks. After micro-plate overnight incubation at 4 °C in order to facilitate sedimentation of the typical integument reddish pigments that could potentially interfere with the measurement, the fluorescence of 7-OHC was determined using micro-plate fluorescence reader (Fluoroskan Ascent, Thermo Scientific, Helsinki, Finland), with 390-nm excitation and 440-nm emission filters. The relative fluorescence units (RFU) were all corrected for background hydrolysis, and nonspecific fluorescence of substrate, and transformed to pmol/minute/integument (activity units) by using a calibration curve constructed with dilutions of 7-OHC in phosphate buffer, run on the same plate containing the samples. Sixteen replicates (each containing six integuments) and their respective blanks were assayed.

### Gene expression by qRT-PCR

One-week-old nymphs were fed *ad libitum* and one week later were dissected and their total RNA extracted from pooled insect tissues (3–4 integuments, 3–4 fat bodies, 5 midguts, and 5 malpighian tubules/pool) with a rotor–stator homogenizer (Glas-Col, Terre Haute, USA) by using the RNAeasy Mini kit (Qiagen, Hilden, Germany) with an on-column DNA digestion step (DNAse I, Qiagen). The RNA was quantified by a NanoDrop^TM^ spectrophotometer (Thermo, Wilmington, USA), and its integrity assessed on a 1% (w/v) agarose gel electrophoresis. Single-strand cDNA was synthesized from 2 μg of total RNA using the High-Capacity RNA-to-cDNA Kit (Applied Biosystems, Carlsbad, USA). The cDNA was then amplified using a Fast SYBR Green Master Mix (Applied Biosystems) in a StepOnePlus Real-Time PCR system (Applied Biosystems) with the universal thermal cycling condition provided by the manufacturer. In order to confirm that only single products were amplified, a temperature-melting step was then performed. Negative controls were performed by using templates generated without reverse transcriptase. Reactions containing primer pairs without template were also included as blank controls. The assay was done in duplicate for each of the three/four independent biological replicates performed. Standard curves were obtained to evaluate the PCR efficiency of each primer pair used, and the comparative Ct (ΔΔCt) method was employed for relative quantification. Both the statistical analysis and the expression plots were done with the REST software (version 2009, Qiagen)^43^. Both β-actin and 18 S RNA were used as housekeeping genes. Primers used are detailed in Table S1.

### Copy number assay by qPCR

Genomic DNA was extracted from S and R nymphs by using the DNAeasy Mini kit (Qiagen, Hilden, Germany). Twenty five nanograms of gDNA was used as template and amplified with the same primer pairs listed in Table S1 for JAR98719, JAR98714 and the housekeeping genes, by using the Fast SYBR Green Master Mix (Applied Biosystems) in a StepOnePlus Real-Time PCR system (Applied Biosystems), with the following cycling conditions: 95 °C for 10 min for initial denaturation and enzyme activation, followed by 40 cycles each of 95 °C for 15 s and 60 °C for 2 min. Relative quantification was performed using the StepOne software version 2.3 (Applied Biosystems), following the comparative ΔΔCT method. For each insect population, three independent biological replicates, each run in triplicate, was assayed.

### Gene knockdown by injecting double-stranded RNA

The dsRNA probe sequences corresponding to the genes *CYP3093A11*, *CYP4EM10*, and methoprene-tolerant (*met*) were amplified by PCR using the set of primers containing T7 promoter sequences listed in Table S1. The *met* gene encodes for the juvenile hormone receptor and was used as a positive control of the silencing procedure, confirming the presence of “adultoids” phenotypes in *T*. *infestans* similar to those described in *R*. *prolixus* by Villalobos-Sambucaro *et al*.^44^: nymphs showing wings and genitalia with abnormal development. These PCR products were used to synthesize dsRNA with the Megascript RNAi kit (Ambion, Austin, USA) according to the manufacturer’s recommendations. The quality and size of the dsRNA products were verified by 1% (w/v) agarose gel. Starved three-week-old fourth instar nymphs (R) were intra-abdominally (ventral) injected with 1 µl of dsRNA solution at 1 µg/µl for each gene, dsCYP3093A11, dsCYP4EM10 or dsMet. Six days after, a group of injected insects were selected randomly and the gene silencing efficiencies were checked out by qPCR as detailed above. The remaining insects were used one week later to check phenotypes after deltamethrin topication (see below). The negative control groups were equally injected with the non-related dsRNA provided with the kit.

### Deltamethrin bioassays

Insects (R) were treated by topical application of the corresponding lethal dose 50% (LD_50_) of deltamethrin in acetone solution on the ventral abdomen of insects, according to the protocol for evaluating insecticide effect on triatomines^45^. The control groups were treated with acetone. Both deltamethrin-treated and control insects were used for gene induction and mortality experiments, as follows: Three and 72 h after topical application, integuments were obtained from R nymphs and the expression patterns of both *CYP3093A* and *CYP4EM* expansions were assayed by qPCR. The time periods were selected based on expression data of other deltamethrin-inducible *CYP* genes in *T*. *infestans* fat body^13^. Three independent replicates of each test, with 3–4 integuments per replicate, were performed. For phenotype assays, mortalities were recorded at 24, 48, and 72 h after topical application. Three independent replicates of each test were performed, with 7–8 insects per group.

### Data Availability

The datasets analysed during the current study are available in the VectorBase repository, https://www.vectorbase.org/organisms/rhodnius-prolixus, and in the GenBank repository, https://www.ncbi.nlm.nih.gov/bioproject/ (BioProjects PRJNA238208 and PRJNA314811).

## Electronic supplementary material

Supplementary Information
