# Supplementary material for: Integument CYP genes of the largest genome-wide cytochrome P450 expansions in triatomines participate in detoxification in deltamethrin-resistant Triatoma infestans
Source: Sci Rep. 2018 Jul 5;8:10177. doi: 10.1038/s41598-018-28475-x (PMC6033900; doi:10.1038/s41598-018-28475-x)
Supplement: Supplementary file 1 — Supplementary Information [file 41598_2018_28475_MOESM1_ESM.pdf]

Supplementary information

Integument CYP genes of the largest genome-wide cytochrome P450 expansions in triatomines participate in detoxification in deltamethrin-resistant *Triatoma infestans*

Andrea B. Dulbecco, Débora E. Moriconi, Gustavo M. Calderón-Fernández, Soledad Lynn, Andrés McCarthy, Gonzalo Roca-Acevedo, Jhon A. Salamanca-Moreno, M. Patricia Juárez and Nicolás Pedrini

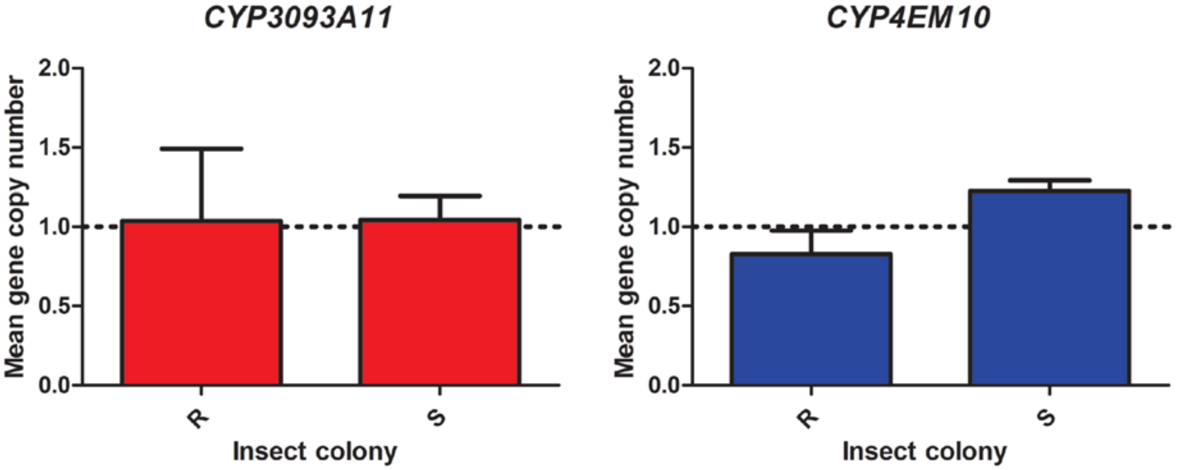

Figure S1. Comparison of the estimated gene copy number of *CYP3093A11* and *CYP4EM10* in genomic DNA of both resistant (R) and susceptible (S) *Triatoma infestans*. The Copy Number Variations (CNV) assay was performed by qPCR as detailed in Methods. For each insect population, three independent biological replicates, each run in triplicate, was assayed. The data presented are the mean *CYP* copy number with their corresponding error bars.

Table S1. Oligonucleotides used in this study.

**Name**	**Forward (5'-3')**	**Reverse (5'-3')**
qPCR		
18S (housekeeping)	GGCGGGGGCATTCGTATTG	ATCGCTGGCTGGCATCGTTTAT
β-actin (housekeeping)	CACCCCAGCAATGTATGTAG	ACCATCAGGAAGTTCGTAAG
JAC17131	TATCTTCTTCCCAGCGCCTTCCA	AGTGAAACTGCCATGGGAACTGA
JAS02904	ATGCGACTTTATAGCCCACCTGC	TTTGGCACACTCTTCTGGCAGA
JAR98714	TGCCACTGAGTTCTAAATCCTG	GAAGCAATCCAGACATTCAGG
JAS02894	CCGGAAGTAATGCTGTTTGATCC	AAAGTTTCCGCCTATGATGCCA
JAS02902	GCCACAAGAGACAAACTTTCCG	TTGGTGCCACTTCAGGATCTTC
JAS02889	AAATGGCGATGGACTGACCG	ATCTCTTCCAGACATCGCTCCTG
JAS02888	ATACGTCCAATCGCCATGCTTC	TTTCGTCGCCGGGTTGGATAC
JAR98719	AAGGGAAGCGACCGGAAGATTG	GATAGCTGTTCCCAAGTTGGTGC
JAS02890	TCAGTGCTGGTCCCAGGAGTTG	AGGACGGCGTGGTTCAATTTG
JAR98713	CTTGGATATCAGGGAAGAAGTGGA	TTTCAAGTTAGGTGGGCGATCA
JAR98715	CACGATGTAGGTAAAGGTTCTGCA	ACATGAACATGCAACACTTTACGC
JAC16640	TAAAGACGGCAGTCTCTGCAGC	GAATACGCTTCGGCAGGCTC
JAC16639	AGCCCACCATCTATCTACGCAG	CTGGACGTGACCATAAGGTTGG
JAC16638	ATCCGTGCGGCTAATATGACTG	TAATGTGTGTCGGGTTGCCAAG
JAC17089	AATGGCATCATAGGCGGAAAC	CATTGTTGCCATCACTGTCCTG
RNAi		
dsCYP3093A11	TAATACGACTCACTATAGGGAGAAG AACAATTAGATATTTTAGGA	TAATACGACTCACTATAGGGAGAGGATAGAATTCAT GTGGATGTGTCCA
dsCYP4EM10	TAATACGACTCACTATAGGGAGAGA TTGCTTCCAAGTTGGTACAG	TAATACGACTCACTATAGGGAGATGGACAGGAATTT CATAAATGC
dsMet	TAATACGACTCACTATAGGGAGATG GAAGGAATGAGAAGAACACG	TAATACGACTCACTATAGGGAGACCATTCTTACCTTC GTTGCTTC
RACE		
3'JAR98719 outer	GAGAGGCTAAAGCTTGGAGAGA	
5'JAR98719 outer	TAGCCGTACACCCTTTGGGA	
3'JAR98719 inner	CGGAAGATTGGCTACAGACT	

5'JAR98719 inner	TGCCACTTCAGGATCATCTC	
3'JAR98714 outer	AGACGAGCATTTCTGGACAGTTT	
5'JAR98714 outer	ATTGCTTCCAAGTTGGTACAGTGC	
3'JAR98714 inner	GCACTGTACCAACTTGGAAGCAAT	
5'JAR98714 inner	CAAACTGTCCAGAAATGCTCGTCT	
